# Analysis of macroautophagy related proteins in G2019S LRRK2 Parkinson’s disease brains with Lewy body pathology

**DOI:** 10.1016/j.brainres.2018.07.023

**Published:** 2018-12-15

**Authors:** Adamantios Mamais, Claudia Manzoni, Iqra Nazish, Charles Arber, Berkiye Sonustun, Selina Wray, Thomas T. Warner, Mark R. Cookson, Patrick A. Lewis, Rina Bandopadhyay

**Affiliations:** a Reta Lila Weston Institute of Neurological Studies, UCL Institute of Neurology, 1 Wakefield Street, London WC1N 1PJ, United Kingdom; b Cell Biology and Gene Expression Section, Laboratory of Neurogenetics, NIA, NIH, Building 35, 35 Convent Drive, Bethesda, MD 20892-3707, USA; c School of Pharmacy, University of Reading, Whiteknights, Reading RG6 6AP, United Kingdom; d Department of Neurodegenerative Diseases, UCL Institute of Neurology, Queen Square, London WC1N 3BG, United Kingdom; e Department of Clinical and Movement Neuroscience, UCL Institute of Neurology, WC1N 3BG, United Kingdom

**Keywords:** LRRK2, Autophagy, p62, LAMP1, LC3, ULK1, Parkinson’s

## Abstract

*LRRK2*, the gene encoding the multidomain kinase Leucine-Rich Repeat Kinase 2 (LRRK2), has been linked to familial and sporadic forms of Parkinson’s disease (PD), as well as cancer, leprosy and Crohn’s disease, establishing it as a target for discovery therapeutics. LRRK2 has been associated with a range of cellular processes, however its physiological and pathological functions remain unclear. The most prevalent LRRK2 mutations in PD have been shown to affect macroautophagy in various cellular models while a role in autophagy signalling has been recapitulated *in vivo*. Dysregulation of autophagy has been implicated in PD pathology, and this raises the possibility that differential autophagic activity is relevant to disease progression in PD patients carrying LRRK2 mutations. To examine the relevance of LRRK2 to the regulation of macroautophagy in a disease setting we examined the levels of autophagic markers in the basal ganglia of G2019S LRRK2 PD post-mortem tissue, in comparison to pathology-matched idiopathic PD (iPD), using immunoblotting (IB). Significantly lower levels of p62 and LAMP1 were observed in G2019S LRRK2 PD compared to iPD cases. Similarly, an increase in ULK1 was observed in iPD but was not reflected in G2019S LRRK2 PD cases. Furthermore, examination of p62 by immunohistochemistry (IH) recapitulated a distinct signature for G2019S PD. IH of LAMP1, LC3 and ULK1 broadly correlated with the IB results. Our data from a small but pathologically well-characterized cases highlights a divergence of G2019S PD carriers in terms of autophagic response in alpha-synuclein pathology affected brain regions compared to iPD.

## Highlights

• Macroautophagy related proteins were analyzed in G2019S LRRK2 PD brains.
• We used four G2019S PD post-mortem brains and pathology matched idiopathic PD cases.
• Lower p62 and LAMP1 levels were observed in G2019S LRRK2 compared to idiopathic cases.
• LRRK2 PD has a divergent autophagic signature to idiopathic Parkinson’s disease.

## 1 Introduction

Coding mutations in *LRRK2* gene on chromosome 12 are associated with familial Parkinson’s disease, with non-coding variation at the *LRRK2* locus also linked to idiopathic Parkinson’s (reviewed in Paisan-Ruiz et al., 2013). While the role of LRRK2 in disease remains unclear, a number of different cellular functions have been attributed to the protein (Wallings et al., 2015). LRRK2 has been implicated in vesicular trafficking pathways, endocytosis and mitochondrial homeostasis, with recent studies suggesting that mutant LRRK2 can alter cellular macroautophagy signaling (Wallings et al., 2015). In terms of the wide range of proposed LRRK2 functions, it is not yet known if these should be considered as cell/tissue specific associations, and which are relevant to the disease process of PD (Lewis and Manzoni, 2012).

Aberrant protein aggregation is a common theme in neurodegenerative diseases, and regulation of protein degradation by autophagic/lysosomal processes has been implicated in the neuropathology of these disorders. Alpha-synuclein (α-Synuclein), a central player in the etiology of PD and the predominant constituent of Lewy bodies, is degraded by macroautophagy and chaperone-mediated autophagy in neurons (Vogiatzi et al., 2008). Cytoplasmic α-synuclein inclusions impair macroautophagy and are resistant to degradation in culture (Tanik et al., 2013). In turn, α-synuclein carrying the PD mutation A53T has been shown to induce mitophagy in neurons mediating toxicity (Choubey et al., 2011). Additionally, the lysosomal markers LAMP2A and hsc70 are reduced in the *substantia nigra* (*s.nigra*) pars compacta and amygdala of PD brains compared to age-matched controls suggesting that autophagy dysregulation is concomitant with α-synuclein pathology in PD (Alvarez-Erviti et al., 2010). This highlights the potential of targeting autophagy to restore physiological α-synuclein turnover as a therapeutic approach (Rubinsztein et al., 2012).

Autophagy is the key cellular process mediating digestion of damaged organelles and proteins as well as the content of vesicles following endocytosis and phagocytosis by lysosomal proteases. Macroautophagy, the most studied form of autophagy, involves the delivery of cargo to lysosomes via double-membrane phagophores and is initiated by phosphorylation events on factors of the ULK1 (Unc Like Kinase 1) complex (Mizushima, 2010). Cellular stress including nutrient starvation, low ATP or heat-shock can induce inhibition of the mTOR complex and activation of AMPK. These changes modulate the phosphorylation state of ULK1 thus mediating its activation (Kim et al., 2011), resulting in induction of macroautophagy initiated by membrane nucleation and autophagosome formation. LC3 (microtubule-associated protein1A/1B light chain3) is a commonly used cellular marker for macroautophagy, with cytosolic LC3-I lipidated to LC3-II and recruited to autophagosomal membranes where it serves as a receptor for selective macroautophagy. LC3-II forms a complex with cargo proteins including p62, which in turn is involved in recruiting substrates to the nascent autophagosome mediating degradation (Klionsky et al., 2016).

A substantial body of literature suggests that LRRK2 is involved in the regulation of autophagy both *in vivo* and *in vitro* (Dodson et al., 2014, Tong et al., 2012, Lynch-Day et al., 2012). LRRK2 is expressed in several different tissues and cell types and within the mammalian brain LRRK2 has higher expression in dopamine innervated regions (Galter et al., 2006). Immunocytochemical and cell fractionation studies suggest that LRRK2 is predominantly cytosolic, but can also be found to be associated with membranes of synaptic vesicles, endosomes and lysosomes at least in the rodent brain (Biskup et al., 2006). Ferree and co-workers, using a systems biology approach, observed that genes linked to autophagy exerted a strong impact on dopaminergic neuron survival in *c.elegans* overexpressing WT-LRRK2 (Ferree et al., 2012). Stimulation of TLR4 receptor or inhibition of mTOR using rapamycin resulted in translocation of LRRK2 to membranes and association with autophagosomes in immune cells (Schapansky et al., 2014). Knockdown of endogenous LRRK2 decreased autophagic flux in monocytes (Schapansky et al., 2014), and loss of LRRK2 in mice results in abnormal kidney development with accumulation of the autophagy markers LC3-II and p62. This was accompanied by alterations in protein degradation, accumulation of α-synuclein and protein carbonyls, and apoptotic cell death (Tong et al., 2010, Tong et al., 2012). It has previously been shown that inhibition of LRRK2 kinase activity using specific inhibitors modulates macroautophagy in cells (Manzoni et al., 2013a, Manzoni et al., 2016, Saez-Atienzar et al., 2014) and that pathogenic mutations in the ROC-COR-kinase domains causes alterations in autophagy and lysosomal markers in patient fibroblasts (Manzoni et al., 2013b, Pedro et al., 2013). In a separate study, it was demonstrated that dopaminergic neurons differentiated from sporadic and familial LRRK2 PD iPSc showed accumulation of autophagic vacuoles (Sánchez-Danés et al., 2012). Therefore, it is quite plausible that LRRK2 is involved in PD pathogenesis through its effect on cellular autophagy pathways (Manzoni and Lewis, 2017). Whether autophagy is altered in the context of LRRK2 mutations in human brain tissue has not, to date, been investigated.

The aim of this study is to test whether alterations in macroautophagy observed in cellular models upon manipulation of LRRK2 are mirrored by changes in autophagy/lysosomal markers in brain tissue from patients with LRRK2 associated PD. To this end we have examined levels of macroautophagy markers in post-mortem tissue from the basal ganglia of PD patients carrying the LRRK2 G2019S mutation and compared that with pathology-matched idiopathic PD and control tissue using immunoblot (IB) and quantitative reverse transcription polymerase chain reaction (qRT-PCR). Cellular expression was established using immunohistochemistry (IH). We observed significantly lower p62 levels in G2019S PD samples compared to iPD and a trend of lower LC3II levels. ULK1 protein levels showed variation with significantly greater levels in iPD compared to control cases, while this was not reflected in the G2019S samples. Significantly lower levels of the lysosomal marker LAMP1 (lysosomal associated membrane protein1) were observed in G2019S PD compared to iPD. The qRT-PCR and IH data of the 4 autophagy related markers are broadly in line with the IB data which reflect alterations in autophagy processes in G2019S PD brains compared to iPD, and provide support for a possible role of LRRK2 in dysregulation of protein degradation and pathology.

## 2 Results

### 2.1 Protein levels of macroautophagy markers

**Fig. 1:**
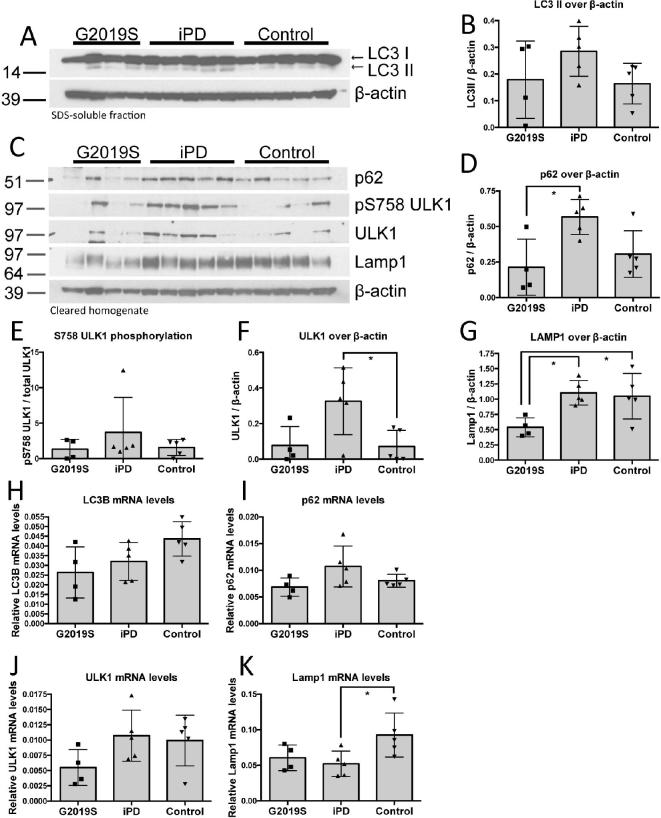
Autophagic markers are altered in G2019S PD brain. Levels of autophagic markers (5% SDS supernatant) were analysed in G2019S PD basal ganglia and compared to pathology-matched iPD and controls. LC3-II showed high variance and no significant divergence in G2019S compared to iPD and controls (A, B). p62 showed significantly higher levels in iPD compared to G2019S PD and a trend towards higher levels compared to controls (C, D). ULK1 levels were increased in iPD compared to controls (C, F) while LAMP1 was significantly lower in G2019S PD samples compared to iPD and controls (C, G). Statistical analysis was performed using ANCOVA with PMD as a covariate (ANCOVA; ^*^p < 0.05; n = 4 cases: G2019S PD; n = 5 cases: iPD, n = 5 cases: Controls; LC3II/actin, F(2,10) = 1.8, P = 0.215; pULK1/ULK1, F(2,10) = 2.1, P = 0.174; p62/actin, F(2,10) = 5.5, P = 0.025; ULK1/actin, F(2,10) = 5.7, P = 0.023; LAMP1/actin, F(2,10) = 4.7, P = 0.037). Relative mRNA levels of the 4 autophagy markers from basal ganglia were measured using qRT-PCR are shown (H-K).

In order to investigate whether autophagy is dysregulated in G2019S PD brain, we compared tissue from four G2019S PD cases to five pathology-matched iPD and five control cases. Tissue from the basal ganglia was used that is impacted by dopaminergic nerve terminal loss and α-synuclein pathology in PD. We examined the relative levels of four autophagy-markers as an indication of steady state autophagic activation. To control for a possible effect of post-mortem delay (PMD) on the levels of these markers, one-way ANCOVA using PMD as a covariate was used for the statistical analysis. The cumulative demographic details of these cases are presented in Supplementary Table S1 and are identical to the cases reported before (Mamais et al., 2013). The processed/lipidated form of LC3, LC3II, was examined in the SDS-soluble fractions produced by differential fractionation, as that is predominantly enriched for membrane-attached moieties. LC3II levels did not differ significantly between G2019S, iPD and control cases when controlling for PMD (Fig. 1A, B). The G2019S PD cases displayed higher LC3 variance with levels similar to control cases.

p62 is a ubiquitin-binding scaffold protein that is degraded through autophagy and plays a role in tagging cargos for degradation (Bjørkøy et al., 2009). p62 levels were found to be significantly upregulated in iPD but not in G2019S PD, when compared to control cases after controlling for PMD (Fig. 1C, D). This may indicate accumulation of p62 in iPD reflecting an impairment in autophagy. Furthermore, these data may also suggest an upregulation of macroautophagy and increase in substrates tagged for degradation by p62, as a response to neurodegeneration, which would be consistent with the trend towards increased LC3-II in the iPD samples.

The kinase ULK1 is a key regulator of the induction of macroautophagy, and is phosphorylated at S758 by mTOR under conditions of nutrient sufficiency, acting to inhibit the activity of this kinase and thereby the induction of autophagy (Kim et al., 2011). No significant alterations were seen in S758 phosphorylation of ULK1, while total ULK1 levels were significantly increased in iPD but not in G2019S PD, when compared to control cases (Fig. 1C, E, F).

LAMP1 is a lysosomal transmembrane protein involved in lysosomal integrity and acidification of the lysosomal lumen. Newly synthesized LAMP1 is transported to lysosomes and endosomes where it regulates lysosomal motility and lysosome-phagosome fusion (Saftig and Klumperman, 2009). LAMP1 showed a significant decrease in G2019S PD cases compared to idiopathic PD, after controlling for PMD (Fig. 1C, G).

### 2.2 mRNA levels of autophagy proteins

We also analysed mRNA transcript levels of the four autophagy markers p62, Lamp1, LC3 and ULK1 in tissue from the basal ganglia by qPCR. We observed varied expression levels across the three sample cohorts and a general trend parallel to the protein findings. LC3B did not show a difference between the sample groups while p62 showed a trend towards an increase in iPD compared to G2019S and control samples which reflects our findings on protein analysis (Fig. 1E, I). ULK1 mRNA levels showed a wide spread across cases with a trend of lower levels in G2019S cases while LAMP1 expression was significantly lower in iPD compared to control samples with a similar but not significant trend in G2019S samples (Fig. 1J, K). The divergence we observe between the trends in protein and mRNA transcript levels in the autophagic markers analysed may be due to inherent differences and limitations of the corresponding techniques, different effect of storage conditions in the integrity or mRNA and protein and lastly actual biological differences between transcript and protein abundance (Maier et al 2009) that in our case may reflect post-translational modifications that modulate protein stability and degradation.

### 2.3 Immunostaining of the macroautophagy markers p62, LAMP1, LC3, ULK1

Our data support a possible alteration of the autophagy flux in LRRK2 PD. The divergent autophagic signature detected in G2019S PD compared to idiopathic PD cases is interesting considering the cases have been matched for pathology severity. Therefore, we next sought to compare the cellular distribution of the autophagic markers LAMP1, LC3, ULK1 and p62 in controls, iPD and G2019S-LRRK2 PD in brain regions affected by α-synuclein pathology using IH. The antibodies used here have all been tested in mammalian cells for cellular localization (Licon-Munoz et al., 2017, Ahmed et al., 2010, Schmitz et al., 2016, Liu et al., 2018). Our negative controls in which either primary or secondary antibody was omitted gave no appreciable immunopositivity on tissue sections.

**Fig. 2:**
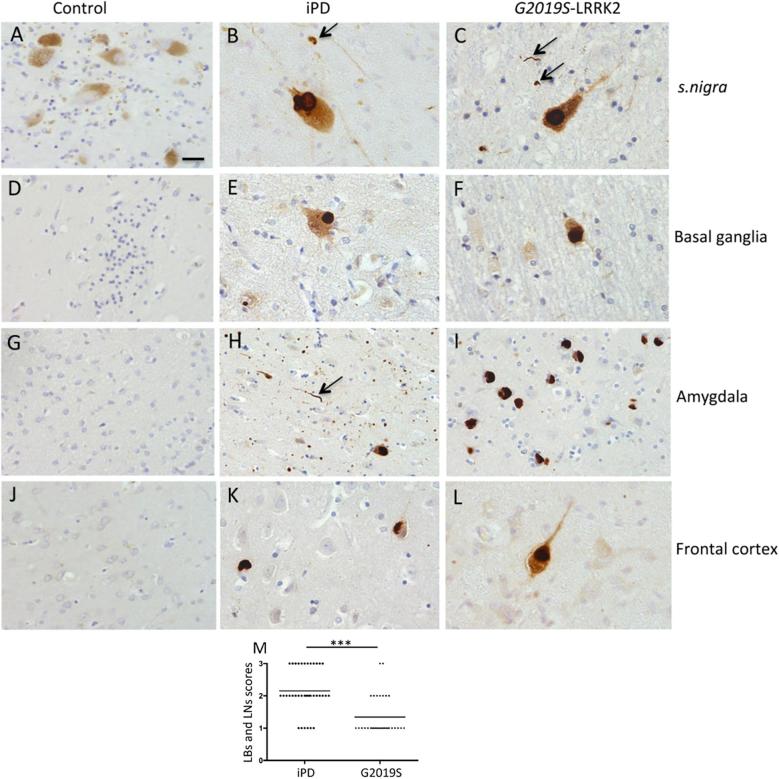
Regional p62 inclusions in control, iPD and G2019S LRRK2 cases as determined by immunohistochemistry. p62 immunopositive LBs were found both in iPD and G2019S cases but not in controls (A-C). Some oligodendroglial inclusions (Black arrows) were immunopositive for p62 in the nigra (B, C). p62 deposits were also noted in medium spiny neurons in basal ganglia of both iPD and G2019S cases (E, F). In amygdala, extracellular p62 granular deposits were noted (H) and abundant cortical type LBs seen in a G2019S case (I). Cortical LBs immunopositive for p62 were also observed in frontal cotex of both PD subgroups (K, L). For all the regions, control cases remained negative for p62 aggregates (A, D, G, J). Scale bar represents 10 μm in all except it is 30 μm in H. Consensus read outs of LB and LN scores in four regions (combined) analysed in iPD and G2019S cases (^***^ denotes P < 0.001 Mann-Whitney *U* test) with the lines representing the mean scores.

2.3.1 p62 IH: In formalin-fixed tissue, we detected moderate to high numbers of pale bodies (PBs) and Lewy bodies (LBs) positive for p62 in both iPD and G2019S LRRK2 mutation cases (Fig. 2). A higher number of p62 positive dystrophic neurites, Lewy neurites (LN) were noted in the iPD cases. Occasional oligodendroglial inclusions immunopositive for p62 were detected in the *s. nigra* of both G2019S PD and iPD. Neuronal cytoplasmic staining was present in the majority of dopaminergic neurons in iPD and G2019S LRRK2 cases. In contrast, control cases did not show p62 immunopositivity in the melanized dopaminergic neurons. The p62 positive inclusions were similar in appearance in both PD cohorts. In basal ganglia and amygdala, higher numbers of p62 positive extracellular deposits were noted in iPD compared to G2019S cases. We detected p62-positive granulovacuolar degeneration bodies GVD’s in the amygdaloid cortex of both affected cohorts with higher numbers presented in G2019S PD. Small numbers of p62 positive neurofibrillary tangles and oligodendroglial inclusions were also observed in some cases, which is likely to be incidental pathology. Semiquantitative scores of p62 inclusions are summarised in Table 1. The combined scores of LBs and LNs from all four regions were significantly higher in iPD compared to G2019S cases (P < 0.001 Mann-Whitney U) (Fig. 2M).

**Table 1:** Summary of p62 staining.

Cases	Regions	Types of p62 inclusions/staining					
		PBs/LBs	dystrophic neurites and LN’s	Neuronal cytoplasmic staining	Extracellular granular staining	GVDs	Coiled bodies and NFTs
Control cases 1–5	s. nigra	–	–	–	–	–	+
	Basal ganglia	–	–	− to +	− to +	–	–
	Amygdala	–	–	− to +	–	–	–
	Frontal cortex	–	–	− to +	–	–	+
iPD cases 1–5	s. nigra	++ to +++	++ to +++	+ to +++	++ to +++	–	+
	Basal ganglia	++ to +++	++ to +++	− to ++	++	–	–
	amygdala	++ to +++	++ to +++	− to ++	++ to +++	++	+
	Frontal cortex	+ to ++	+ to ++	− to ++	+ to ++	+	+
G2019S cases 1–4	s. nigra	+ to +++	+ to ++	+ to +++	+ to ++	–	+
	Basal ganglia	+ to ++	+	− to ++	+	–	–
	Amygdala	+ to +++	+	− to ++	+ to ++	+ to +++	–
	Frontal cortex	+ to ++	+	− to +	+ to ++	+ to ++	+

Key: − (no inclusions/staining); + = occasional inclusions/staining ; ++ = moderate inclusions/staining ; +++ = large number of inclusions/.

PBs = Pale bodies; LBs = Lewy bodies, LNs = Lewy neurites; NFTs = Neurofibrillary tangles.

**Fig. 3:**
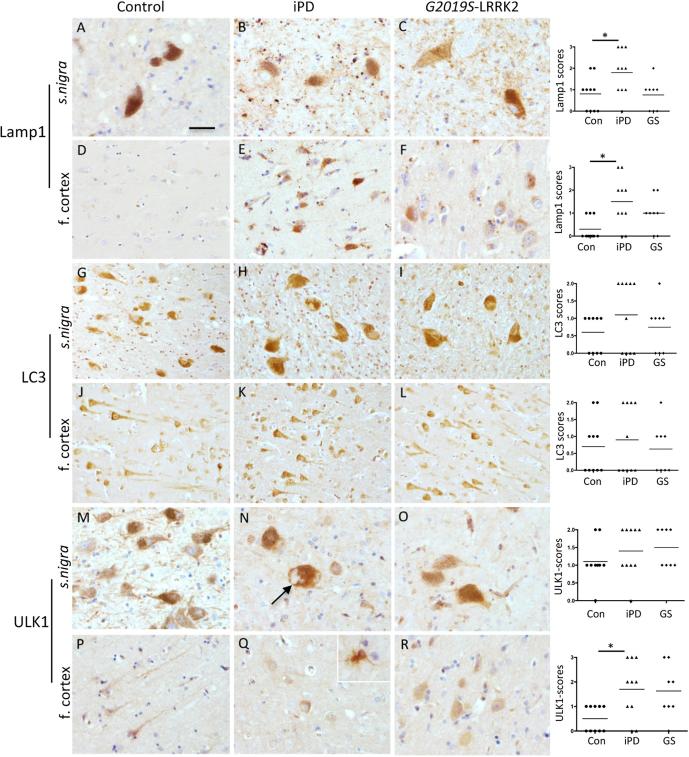
Representative images of immunohistochemistry of LAMP1, LC3 and ULK1 from *s.nigra* and frontal cortex of controls, iPD and G2019S_LRRK2 PD postmortem brains. A-F is Lamp1; G-L is LC3; M-R is ULK1. The scale bar is 20 μm in A, B,C, F, M, N, O and R and 40 μm in D-L, and P, Q. Black arrow in N points to *s.nigra* LBs negative for ULK1. Inset in Q shows white matter glia positive for ULK1. Corresponding semiquantitative IH scores from neurons and glia from controls, iPD and G2019S cases are shown for s. nigra and frontal cortex (f.cortex). Graphs represent consensus scores for each autophagy marker with the line representing the mean scores. Statistical significance of scores between the case groups were tested using Kruskall-Wallis statistic with Dunn’s post-hoc test (^*^ denotes P < 0.05).

2.3.2 LAMP-1 IH: demonstrated punctate deposits within some neurons and in glial cells. Majority of glial staining morphologically appeared to associate with microglia. There was some accumulation of cytosolic LAMP-1 immunoreactivity (IR) within dopaminergic neurons in the *s. nigra* in iPD and also in G2019S-LRRK2 cases compared to controls. There was some upregulation of LAMP-1 expression associated with frontal cortical neurons in both the PD subtypes tested. Frontal cortical neurons however expressed higher levels of LAMP-1 compared to controls. Glial cells displayed strong immunoreactivity for LAMP-1 in iPD cases (Fig. 3). Immunohistochemistry scores are presented in Fig. 3. The IH scores were significantly higher for iPD compared to control cases in both *s.nigra* and frontal cortex (Kruskall Wallis test with Dunn’s multiple comparison test). There were no LBs or LNs positive for LAMP-1.

2.3.3 LC3-IH: The antibody recognises both LC3I and II. In all cases examined, substantial population of cortical neurons constitutively exhibited LC3 IR within the cytoplasm at varying intensities. DAergic neurons showed lower LC3 levels. LBs and LNs remained negative for LC3 IR. Some cortical neurons demonstrated higher perinuclear LC3 staining compared to the processes. Glial processes were negative for LC3 IR although some glial nuclei were moderately positive for LC3 IR (Fig. 3). LB’s and LNs were unstained for LC3 in all PD cases examined. Higher LC3 scores were recorded in both *s.nigra* and frontal cortex although these were not statistically significant.

2.3.4 ULK-1IH: ULK1-IR was apparent in the cytoplasm of some neurons from the *s. nigra* and frontal cortex of controls, iPD and G2019S. In controls, ULK-1 IR was also seen in neuronal processes in pyramidal neurones of frontal cortex and also DAergic neurons in the *s. nigra*, which were not observed in PD neurons in PD cases. Reactive astrocytes were positive for ULK-1 IR and were more apparent in the white matter-grey matter border of PD cases (Fig. 3). ULK1 scores in the *s. nigra* were very similar in all three case groups, however in the frontal cortex, ULK1 scores were significantly higher in iPD compared to controls (P < 0.05; Kruskall Wallis test with Dunn’s multiple comparison).

## 3 Discussion

Autophagy comprises a range of highly conserved cellular processes that contribute to protein homeostasis. Autophagic activity decreases with age with dysregulation of macroautophagy and chaperone-mediated autophagy reported in a wide spectrum of tissues and diseases (Cuervo and Dice, 2000, Terman, 1995). In neurodegenerative disease, a number of dysregulated signaling events related to protein turnover and degradation have been reported. Mutations in *PINK1* and *PRKN*, coding for the kinase PINK1 and the ubiquitin ligase Parkin respectively, are associated with autosomal-recessive PD and act to disrupt mitophagy – leading to reduced clearance of damaged mitochondrial and hence cell death (Yamano et al., 2016). Furthermore, a number of studies have proposed that basal levels of autophagy are important for the clearance of α-synuclein, thus limiting intracellular oligomerization and formation of aggregates (Vogiatzi et al., 2008). Interestingly, studies on autophagic markers in the context of neurodegenerative disease namely, Dementia with Lewy bodies (DLB) has shown some alterations compared to controls (Gurney, 2018, Higashi et al., 2011).

In this study, we investigated markers of macroautophagy in brain tissue from iPD, G2019S LRRK2 PD and control cases. Specifically, we examined LC3-II, p62, phosphorylated ULK1 and LAMP1 levels in the basal ganglia of post-mortem brain tissue. Previous work from our group has shown that mutations in LRRK2 lead to dysregulation of the autophagic response to starvation. Fibroblasts from patients carrying the R1441C, Y1699C or G2019S mutations show an attenuated response to macroautophagy activation by starvation compared to WT cells, as indicated by modulation of LC3 I/II levels upon cell starvation (Manzoni et al., 2013b). In this study, we observed a high variance of LC3-II in G2019S and no significant difference to iPD or controls. A larger number of cases would need to be analyzed to yield a more granular understanding of LC3-II changes in G2019S PD.

**Fig. 4:**
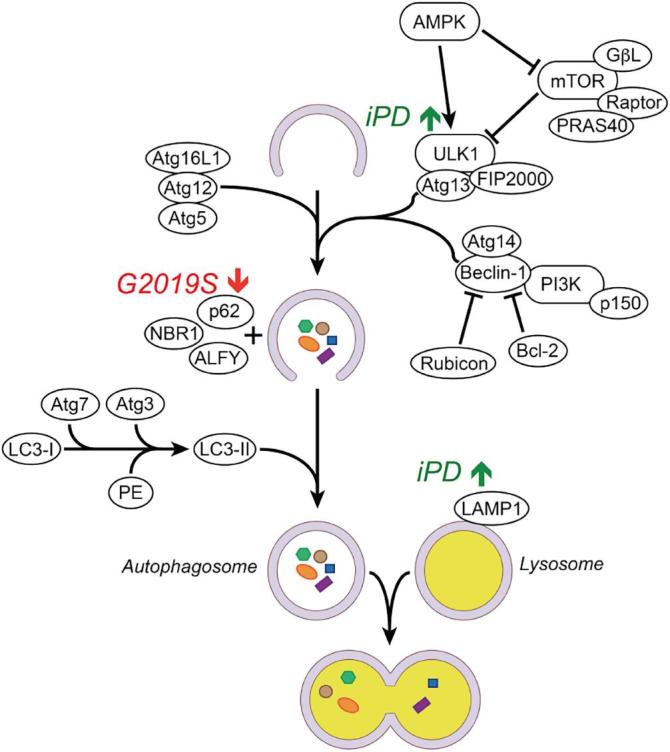
Schematic of macroautophagy activation and changes observed in post-mortem iPD and G2019S PD cases. Upstream AMPK and mTOR -dependent signals mobilise ULK1 and a spectrum of Atg factors as a response to oxidative stress, nutrient starvation and other cellular stresses. Initial stages of autophagosome formation see LC3-II recruitment to the autophagosome membrane followed by docking of p62. This results in autophagosome closure and engulfment of cargoes to be degraded following fusion with lysosomes. Lower levels of p62 observed in G2019S PD compared to iPD may reflect poor p62 production with accumulation of undegraded cargo or abnormal increase in the speed of autophagosome/p62 degradation. LAMP1 levels were increased in iPD compared to G2019S PD suggesting a dysregulation of trafficking events that mediate sequestration of lysosomal components in genetic PD cases.

Our results indicate higher p62 levels in iPD compared to G2019S cases and a trend of higher levels compared to control tissue, reflecting either accumulation of un-degraded cargo or an increase in the basal levels of cargo processing. p62 levels in the G2019S PD samples were similar to controls. We have shown that cells carrying ROC-COR domain mutations (R1441G and Y1699C) show fewer p62-positive puncta following starvation (Manzoni et al., 2013b). In a different study, G2019S PD fibroblasts were reported to have increased basal LC3-II and lower p62 levels compared to control cells (Pedro et al., 2013). The data presented here are consistent with cell studies suggesting that an impairment in macroautophagy as seen in PD (Wang et al., 2016) may be distinct in LRRK2 genetic PD cases. Intriguingly, our data suggest a decrease in LAMP1 levels in the G2019S PD tissue compared to both iPD and pathologically normal control samples, further highlighting a divergence in pathobiology between LRRK2 PD and iPD. These data have been summarized in Fig. 4. Winslow and colleagues have previously shown that α-synuclein can dysregulate Atg9 localization and impair macroautophagy by inhibiting the small Rab GTPase Rab1a (Winslow et al., 2010). We have previously shown divergent solubility and aggregation properties of α-synuclein in the G2019S PD cases examined here compared to pathology-matched iPD cases (Mamais et al., 2013). It is a plausible hypothesis that a difference in the biochemical properties of α-synuclein may underlie a divergence in macroautophagy events accompanying neuropathology. To test this hypothesis we performed a linear regression analysis that showed a significant correlation between LAMP1 levels and aggregated α-synuclein levels in the urea fraction of iPD samples, while the G2019S samples showed a significant correlation of both p62 and ULK1 levels with aggregated α-synuclein (Supplementary Table S2. Lastly, we tested whether disease duration may explain any differences we observe in autophagic markers. A significant inverse correlation between p62 levels and disease duration was observed in iPD while both LC3II and phosphorylated ULK1 levels showed a very strong inverse correlation with disease duration in G2019S cases suggesting that disease severity may correlate with dysregulation of autophagy (Supplementary Table S2). These results overall highlight a divergent autophagic signature of G2019S PD cases that may correlate with synuclein aggregation and examination of further cases and controls is warranted to address this.

To further characterize the autophagic signature of the G2019S PD cases the expression and distribution of p62 was investigated by immunostaining. The iPD and G2019S PD cases were classified as having the limbic sub-type distribution of α-synuclein pathology and were described previously (Mamais et al., 2013). p62 was detected in PBs and LBs in both idiopathic and LRRK2 PD cohorts in the *s.nigra*. Lower numbers of p62-positive Lewy neurites were detected in G2019S PD compared to iPD cases. p62 was also detected in GVDs, which can form as a result of defective autolysosome formation (Funk et al., 2011). Interestingly, higher numbers of p62-positive GVDs were detected in G2019S PD compared to sporadic cases. The immunoblot data from LAMP1, LC3 and ULK1 were consistent with the IH data (Fig. 2). We did not observe any LC3 positive LBs, in contrast to a recent report (Higashi et al., 2011).

The role of LRRK2 in vesicular trafficking has gained substantial interest in recent years. LRRK2 can associate and directly phosphorylate a subgroup of small Rab GTPases, involved in regulating the specificity and directionality of membrane trafficking processes (Steger et al., 2016). LRRK2 associates with Rab29/Rab7L1 mediating trans-Golgi clustering and clearance of trans-Golgi-derived vesicles and this is affected by PD-linked mutations on LRRK2 (Beilina et al., 2014). Furthermore, LRRK2 and Rab29/Rab7L1 control the integrity of the endolysosomal protein degradation process in a common pathway that is affected by PD-linked mutations (Fujimoto et al., 2018, MacLeod et al., 2013, Puriyte et al., 2018, Zhiyong et al., 2018). LRRK2 KO mice display a biphasic alteration of autophagic markers with age, with lower levels of p62 at 7 months old and higher levels at 20 months old compared to WT mice (Tong et al., 2012). It is possible that an effect of LRRK2 on macroautophagy as seen by altered LC3-II or p62 levels is a result of dysregulated targeting of lysosomal components to the autolysosome. The hypothesis of dysregulated lysosomal biogenesis is supported by lysosomal enlargement and a deficit in recruitment of lysosomal hydrolases seen in rat primary neurons expressing G2019S LRRK2 (MacLeod et al., 2013). The lysosomal protein LAMP1 is transported from the trans-Golgi network to lysosomes where it mediates lysosomal maturation, fusion with autophagosomes and degradation of cargo (Saftig and Klumperman, 2009). Phosphorylation of p62 by LRRK2 has been reported recently, and it may provide a context to explain the autophagic differences we observe between iPD and G2019S tissue. According to Kalogeropoulou and colleagues (Kaleogeropulou et al., 2018), LRRK2 mutations induce cellular toxicity synergistically with p62 and dependent on p62 T138 phosphorylation. If LRRK2-mediated phosphorylation of p62 induces p62-mediated macroautophagy this may be reflected by altered protein levels in post-mortem tissue driven by the G2019S hyperactive variant, and would certainly underlie a divergent autophagic signature compared to iPD. Furthermore, mutations in p62 in amyotrophic lateral sclerosis has been identified recently (Teyssou et al., 2013) implying the importance of autophagy regulation in neurodegeneration. Finally, recent data from our group has shown that chronic LRRK2 kinase inhibition imparts mTOR independent alteration in ULK1 phosphorylation in astrocyte cultures further reiterating the importance of autophagy regulation in the context of LRRK2 pathobiology (Manzoni et al., 2018).

The data presented in this study demonstrate decreased levels of LAMP1 in the basal ganglia of G2019S mutation carriers compared to iPD and control cases, and are consistent with a model of impairment of lysosomal targeting in *LRRK2* PD. Collectively, our results support a distinct molecular signature for *LRRK2* PD compared to idiopathic disease, where dysregulation of vesicular trafficking and sequestration of lysosomal components underpins alterations in macroautophagy and protein clearance. A significant caveat to these conclusions is the limited number of samples available for inclusion in this study. Although care was taken to match genetically defined mutation carrier samples to idiopathic cases and controls, as demonstrated by the extensive statistical analysis undertaken (Supplementary Tables S1 and S2), it is not possible to eliminate or completely control for the intrinsic variability observed in human pathological samples. Analysis of further cases, therefore, and in particular from patients with *LRRK2* mutations other than the G2019S variant, will be critical to provide further resolution to the nature of the changes in autophagy in the brains of individuals with *LRRK2* associated PD. In this context, it is of interest to note that LRRK2 levels are increased in early stages of iPD (Dzamko et al., 2017), but are reduced in LRRK2 mutation cases (Zhao et al., 2018). It is also important to note that this study analyzed the situation in the brain *post mortem*, and is therefore unavoidably static in nature – precluding the possibility of an examination of autophagic flux. As such, and due to the dynamic nature of macroautophagy, it is not possible to discriminate between alterations in induction of macroautophagy and changes in fusion with the lysosomes and recycling of autophagic vesicles. To achieve this, further investigations in animal and cellular models for LRRK2 function will be required.

## 4 Experimental procedure

### 4.1 Case details and tissue collection

The post-mortem human brain tissues used were previously described (Mamais et al., 2013, Khan et al., 2005). All 14 cases were obtained from the Queen Square Brain Bank. Appropriate consent was obtained and approval for this study was granted by the local Research Ethics Committee. Neuropathological classification was of the limbic sub-type for α-synuclein pathology in all G2019S PD cases (McKeith et al., 2005). The cumulated demographics of the cases used are provided in Supplementary Table S1. Alpha-synuclein pathology matched iPD cases were used and the controls had no observable neuropathology or clinically diagnosed neurological disease. All PD cases showed slow progressive parkinsonism with good levodopa response. Flash frozen tissue was obtained from the basal ganglia (caudate and putamen), and formalin fixed tissue blocks from *s.nigra,* basal ganglia, amygdala (part of limbic cortex), and frontal cortex were used for immunohistochemistry. It is important to note that our control and iPD cases investigated here were negative for G2019S mutation.

### 4.2 Differential ultracentrifugation and fractionation

Tissue was processed as described (Mamais et al., 2013, Bandopadhyay, 2016). Briefly, 10% (w/v) homogenates were prepared from 1 g tissue from the basal ganglia in homogenization buffer [20 mM Tris–HCl pH 7.4, 150 mM NaCl, protease and phosphatase inhibitors (ROCHE)] and cleared by centrifugation at 1000*g*, 5 min, 4 °C. The cleared homogenate was centrifuged at 100,000*g*, 1 h, 4 °C to produce the TBS-soluble fraction (supernatant). The pellet was washed and resuspended by sonication in 2.5 ml homogenization buffer supplemented with 5% (w/v) SDS, and centrifuged at 100,000*g*, for 30 min, at 16 °C to produce the SDS-soluble fraction (supernatant).

### 4.3 Antibodies used

The following antibodies: p62 (BD Biosciences 610833), LAMP1 (ABCAM, ab25630), LC3 (Novus, NB100-2220), β-actin (Sigma, clone AC-40), pS758 ULK1 (Cell Signaling, 6888), and ULK1 (Cell Signaling, 8054) were used for both immunoblots and IH protocols. These antibodies are extensively characterized for use in both techniques as mentioned in manufacturer’s websites and some are characterized against the corresponding knock-out tissue.

### 4.4 Immunoblotting

30 μg of each sample were analyzed by western blotting, using standard techniques and as described previously (Mamais et al., 2013). Results of individual autophagy markers are expressed relative to β-actin used as a housekeeping marker. The statistical test used for immunoblots were one-way ANOVA with Tukey’s post-hoc correction as indicated in the text.

### 4.5 qRT-PCR of autophagy markers

Small chunks of basal ganglia were homogenized and RNA was extracted using Trizol reagent (Thermo Fisher), as per manufacturers instructions. RNA concentration and purity was measured spectrophotometrically using a Nanodrop (Thermo scientific). All RNA samples had an A260/A280 ratio above 1.9. 1 μg of RNA was reverse transcribed using superscriptIV (Thermo Fisher) using random hexamer primers. qPCR was performed using Power SYBR green master mix (Thermo Fisher) and a Stratagene Mx3000P cycler (Agilent). The primer pairs used were RPL19a Forward: 5′CCCACAACATGTACCGGGAA3′; RPL19a Reverse: 5′TCTTGGAGTCGTGGAACTGC3′; SQSTM1 (p62) Forward: 5′CCTCCTAACAAGTGTATCTC3′, SQSTM1 (p62) Reverse: 5′ACGACTATGTGACCTCTT3′; LAMP1 Forward: 5′CATACACTCACTCTCAAT3′, LAMP1 Reverse 5′ATGTCAGTTATAGATTCCA3′; ULK1: Forward: 5′GTCACACGCCACATAACAG3′; ULK1 Reverse: 5′TCTTCTAAGTCCAAGCACAGT3′; LC3B: Forward: 5′AGTAGTTATCACTCTTAGG3′; LC3B Reverse: 5′CTCTTGACATTAGTATCTG3′. The cycling protocol consisted of an initial denaturation of 95˚C for 10 min followed by 40 cycles involving denaturation at 95˚C for 30sec followed by annealing for 60sec at 60˚C and extension for 60sec at 72˚C. A dissociation curve was added at the end to confirm a single amplicon. All samples were amplified in duplicates. Data were generated using the deltaCT methods and results were normalized to the reference gene RPL19a and relative expression to one control brain sample (Pfaffl, 2001).

### 4.6 Immunohistochemistry

Standard immunohistochemistry protocols were followed as described previously (Mamais et al., 2013). Briefly, formalin-fixed tissue sections from *s. nigra*, basal ganglia, amygdala and frontal cortex were immersed in methanol containing 0.3% H_2_O_2_ to quench endogenous peroxidase activity and pretreated with pressure cooking in 10 mM citrate buffer at pH 6.0 for 10 min to expose antigenic sites. Sections were then blocked in 10% milk for 1 hr and incubated with mouse monoclonal primary antibody to p62 (BD transduction laboratory) at 1:300; LAMP-1 (1:200); LC3 1:100; ULK1 1:100 dilution, overnight at 4 °C. Following biotinylated secondary antibody (DAKO) administration, the sections were washed and incubated with the avidin-biotinylated enzyme complex and finally visualized with hydrogen peroxide (0.05%) activated 3,3′-diaminobenzidine chromogen. Sections were lightly counterstained with Meyer’s haematoxylin, dehydrated in graded alcohols, cleared in xylene and cover slipped in DPX mounting medium (BDH). All sections were stained twice with similar results. Negative controls by omitting the primary and secondary antibodies were run concurrently for each antibody.

### 4.7 Semi-quantitative scoring of p62 inclusions and IH scores of Lamp1, LC3 and ULK1.

A four-point scoring system was employed to score for number of p62 positive inclusions similar to that employed previously by our group (Mamais et al., 2013, Sharma et al., 2011). Scoring was carried out independently by two researchers (RB and IN) and the consensus results are presented. All p62 scores were done at X20 objective. Score “-/0” is equivalent to absence of staining or no inclusions; “+/1” = occasional staining or up to 2 inclusions; “++/2” = moderate staining or between 3 and 10 p62 inclusions and “+++/3” = large number of neurons showing p62 staining or greater than 10 inclusions counted within the field. Similarly, semiquantitaive assessments of DAB immunohistochemistry for LAMP1, LC3 and ULK1 were done on a 4-point grading scale: 0 indicates no staining (neuronal or glial) that could be considered to be above background level; 1 indicates few cells (2+) (neuronal or glial) lightly stained, 2 indicates that most cells (neuronal and glial) moderately stained and 3 indicates most/all (neuronal or glial) cells strongly stained. Pooled scores from neurons and glia staining were analysed using Kruskall Wallis test followed by Dunn’s multiple comparison analysis.
