# Supplementary material for: Analysis of macroautophagy related proteins in G2019S LRRK2 Parkinson’s disease brains with Lewy body pathology
Source: Brain Res. 2018 Dec 15;1701:75–84. doi: 10.1016/j.brainres.2018.07.023 (PMC6361106; doi:10.1016/j.brainres.2018.07.023)
Supplement: Supplementary data 1 [file mmc1.docx]

| Cases | F:M | Age  (yrs)  Mean + SEM | PMD(h) Mean + SEM | pH of tissue  Mean+SEM | Alpha-synuclein pathology | Storage duration  (yrs)  Mean + SEM |
| --- | --- | --- | --- | --- | --- | --- |
| Control  iPD  G2019S | 3:2  3:2  4:0 | 84.80+4.43  77.80+4.1  79.25+2.56 | 65+16.6  44.09+8.56  29.04+6.22 | 6.21+0.09  6.29+0.10  6.11+0.16 | N/A  limbic  limbic | 6.8+0.58  12+0.55  14+2.74 |

Supplementary Table 1: Selected demographics (cumulated) of cases studied. Age, PMD, pH of tissue did not differ significantly amongst the 3 groups (P>0.05; Kruskall-Wallis test with Dunn’s multiple comparison) More detailed breakdown is provided in Mamais et al 2013. Alpha-synuclein pathology criterion according to McKeith et al 2005.
