# Supplementary material for: Analysis of macroautophagy related proteins in G2019S LRRK2 Parkinson’s disease brains with Lewy body pathology
Source: Brain Res. 2018 Dec 15;1701:75–84. doi: 10.1016/j.brainres.2018.07.023 (PMC6361106; doi:10.1016/j.brainres.2018.07.023)
Supplement: Supplementary data 2 [file mmc2.docx]

| **iPD cases (n=5)** | | | | Basal Ganglia Synuclein | | | |
| --- | --- | --- | --- | --- | --- | --- | --- |
|  | Age | Disease duration (yrs) | Sex (F vs M) | Homo-genates | TBS fraction | SDS fraction | Urea fraction |
| LC3II/Actin | r=0.334, P=0.583 | r=-0.096, P=0.877 | r=-0.535, P=0.352 | r=-0.164, P=0.793 | r=-0.531, P=0.357 | r=-0.585, P=0.300 | r=0.549, P=0.338 |
| P62/Actin | r=0.549, P=0.338 | **r=-0.979, P=0.004** | r=-0.384, P=0.524 | r=0.771, P=0.127 | r=0.716, P=0.173 | r=0.139, P=0.823 | r=-0.538, P=0.350 |
| pULK1/ULK1 | r=0.641, P=0.244 | r=-0.705, P=0.183 | r=-0.376, P=0.533 | r=0.232, P=0.707 | r=0.003, P=0.997 | r=-0.510, P=0.380 | r=-0.234, P=0.705 |
| ULK1/Actin | r=-0.545, P=0.342 | r=0.602, P=0.282 | r=0.014, P=0.982 | r=-0.035, P=0.955 | r=0.064, P=0.918 | r=0.584, P=0.301 | r=0.505, P=0.386 |
| Lamp1/Actin | r=-0.022, P=0.972 | r=-0.154, P=0.805 | r=-0.734, P=0.158 | r=0.157, P=0.801 | r=-0.146, P=0.815 | r=0.060, P=0.924 | **r=0.837, P=0.077** |
|  | | | | | | | |
| **G2019S cases (n=4)** | | | | Basal Ganglia Synuclein | | | |
|  | Age | Disease duration (yrs) | Gender  (F vs M) | Homo-genates | TBS fraction | SDS fraction | Urea fraction |
| LC3II/Actin | r=-0.515, P=0.485 | **r=-0.999, P=0.026** | n/a | r=0.538, P=0.462 | r=-0.017, P=0.983 | r=0.461, P=0.539 | r=0.624, P=0.376 |
| P62/Actin | r=-0.050, P=0.950 | r=-0.652, P=0.548 | n/a | r=0.588, P=0.412 | r=0.355, P=0.645 | r=0.358, P=0.642 | **r=0.938, P=0.062** |
| pULK1/ULK1 | r=-0.638, P=0.362 | **r=-1.000, P=0.001** | n/a | r=0.360, P=0.640 | r=-0.178, P=0.822 | r=0.247, P=0.753 | r=0.573, P=0.427 |
| ULK1/Actin | r=0.072, P=0.928 | r=-0.626, P=0.569 | n/a | r=0.693, P=0.307 | r=0.484, P=0.516 | r=0.477, P=0.523 | **r=0.979, P=0.021** |
| Lamp1/Actin | r=-0.850, P=0.150 | r=-0.885, P=0.308 | n/a | r=0.110, P=0.890 | r=-0.479, P=0.521 | r=0.101, P=0.899 | r=0.187, P=0.813 |
|  | | | | | | | |
| **iPD, G2019S and control cases combined (n=14)** | |  |  |  |  |  |  |
|  | Strorage  duration  (yrs) |  |  |  |  |  |  |
| LC3II/actin | r=0.171, P=0.559 |  |  |  |  |  |  |
| P62/Actin | r=0.170, P=0.561 |  |  |  |  |  |  |
| pULK1/ULK1 | r=0.072, P=0.808 |  |  |  |  |  |  |
| ULK1/actin | r=0.306, P=0.287 |  |  |  |  |  |  |
| Lamp1/Actin | r=-0.377, P=0.184 |  |  |  |  |  |  |

Supplementary Table S2. Pearson’s *r* was calculated to measure the linear correlation between levels of autophagic markers and age, disease duration, gender and α-synuclein protein levels in the basal ganglia of the cases studied (synuclein data previously presented in Mamais et al, 2013). In iPD samples P62 levels showed a statistically significant inverse correlation with age while the G2019S samples showed a significant parallel effect on LC3II levels and ULK1 phospho-levels. Correlation analysis between autophagy markers and gender was not possible in G2019S as all cases were female. A significant correlation was found between levels of urea-soluble aggregated α-synuclein and Lamp1 in the Basal Ganglia in iPD cases, while in G2019S cases aggregated α-synuclein correlated with P62 and ULK1 levels in this brain region. Storage duration of the brain samples was tested for correlation with levels of autophagic markers in iPD, G2019S and control cases showing no apparent correlation (bottom panel).
